# *Schistosoma mansoni*-Associated Morbidity among Preschool-Aged Children along the Shores of Lake Victoria in Uganda

**DOI:** 10.3390/tropicalmed2040058

**Published:** 2017-11-05

**Authors:** Allen Nalugwa, Fred Nuwaha, Edridah Muheki Tukahebwa, Annette Olsen

**Affiliations:** 1 Child Health and Development Centre, College of Health Sciences, Makerere University, P.O. Box 6717 Kampala, Uganda; 2 Disease Control and Prevention, School of Public Health, Makerere University, P.O. Box 7062 Kampala, Uganda; fnuwaha@musph.ac.ug; 3 Neglected Tropical Diseases, Vector Control Division, Ministry of Health, P.O. Box 1661 Kampala, Uganda; edmuheki@gmail.com; 4 Parasitology and Aquatic Pathobiology, Faculty of Health and Medical Sciences, University of Copenhagen, DK-1870 Frederiksberg C, Denmark; aol@sund.ku.dk

**Keywords:** *Schistosoma mansoni*, morbidity, preschool-aged children, Lake Victoria shoreline, Uganda

## Abstract

*Schistosoma mansoni* causes morbidity in human beings, with the highest prevalence in rural sub-Saharan Africa. Prolonged *S. mansoni* infection with egg deposition in intestinal blood vessels leads to liver and spleen enlargement, and thus chronic morbidity. The objective of this study was to assess whether preschool-aged children develop severe *S. mansoni*-related morbidity. Parasitological, clinical, and ultrasonographic examinations were carried out in 916 preschool-aged children in five schistosomiasis-endemic districts (Bugiri, Buikwe, Jinja, Mayuge, and Namayingo) along the Lake Victoria shoreline in east-central Uganda. Anaemia and anthropometry measurements were also taken. Using the Kato-Katz technique on one stool sample collected on three consecutive days, 74.9% (686/916) were found infected with *S. mansoni*; the majority were lightly infected (57.9%), while 22.7% and 19.4% were moderately and heavily infected, respectively. The overall geometric mean intensity (GMI) of infected children was 294.2 eggs per gram faeces. Mayuge and Jinja districts had the highest (51.2%) and lowest (2.2%) number of infected children, respectively. Hookworm infection was found in 7.8% (71/916) of the children. Both liver and spleen were significantly more enlarged in the infected children than in the uninfected children (*p* < 0.0005), as measured by ultrasonography. Physical palpation of the spleen was more often detected in the uninfected children. A significantly (*p* < 0.0005) higher proportion of *S. mansoni*-positive children were anaemic (359/686; 52.3%) compared to the children who had no eggs in their stool samples (81/230; 35.2%). *Schistosoma mansoni* infection did not have any severe effect on the nutrition status of preschool-aged children. Neither infected nor uninfected children were found to be underweight or stunted. Liver fibrosis with distinct Symmer’s ‘pipe stems’ was found in a few heavily-infected children (0.3%). In a linear multivariable regression analysis, age of the child, anaemia, liver fibrosis, and size of the left liver lobe were associated with *S. mansoni* intensity of infection (adjusted R^2^ = 0.11; *p* < 0.0005). Our results demonstrate that *S. mansoni*-related morbidity does develop in children less than six years of age, and that older children (37–60 months) are at higher risk (regression coefficient 0.33; *p* <0.0005) compared to younger ones (12–36 months). We recommend that preschool-aged children be included in the target population for schistosomiasis mass treatment so as to prevent the childhood chronic form of schistosomiasis.

## 1. Introduction

Intestinal schistosomiasis is caused by infections with *Schistosoma mansoni* and presents a significant health burden, particularly in sub-Saharan Africa [1]. Once an individual is infected with *S. mansoni* parasites, they are at risk of developing the related morbidity [2]. *Schistosoma mansoni* is highly endemic in Uganda [3,4,5,6,7], and the associated morbidity—particularly liver fibrosis—is common in the infected communities [8]. Schistosomiasis morbidity is caused primarily by detrimental immunologic responses to *S. mansoni* eggs deposited in the liver and intestines by adult female worms living in the blood vessels surrounding these organs [8,9].

Morbidity associated with *S. mansoni* such as hepatosplenomegaly, dilation of the portal vein, anaemia, decreased physical fitness, and delayed physical and cognitive development has been characterised in school-aged children [9,10,11] and in adults [7,8,12]. Limited data is available on schistosomiasis-associated morbidity in preschool-aged children aged less than six years, where infection with *S. mansoni* was initially thought to be rare [8,13]. However, recent data suggest that children less than six years of age are a high-risk group for *S. mansoni* infection [5,14]. A study of morbidity associated with *S. mansoni* in preschool-aged children is interesting as the infection in such children is likely to be contracted recently, thereby providing an opportunity to assess the early effects of infection with *S. mansoni*.

In this community-based study we describe anthropometric indices, haemoglobin levels, and the sizes of liver, spleen, as well as the portal vein diameter among children aged 12–60 months infected or not infected with *S. mansoni* living along the Lake Victoria shoreline, eastern Uganda. This description can be used as a baseline for evaluating the effect of *S. mansoni* control measures on these indices.

## 2. Materials and Methods

### 2.1. Study Area and Participants

The study was carried out in *S. mansoni* endemic communities along the north-east shoreline of Lake Victoria in eastern Uganda from April 2013 to February 2014. The study area has been described in detail elsewhere [5], but in brief, five districts (Bugiri, Buikwe, Jinja, Mayuge, Namayingo) were surveyed and a random sample of 35 fishing communities was selected using a table of random numbers. All children aged 12 to 60 months who were present on the days of survey were included in the study and investigated for all parameters, irrespective of their *S. mansoni* infection status.

### 2.2. Parasitological Examination

Each child provided one stool sample on three consecutive days, and the samples were processed according to the modified Kato-Katz thick smear technique [15] using 41.7 mg templates. Two slides were prepared from each stool specimen and examined under a microscope (10× magnification) within 30 min to detect hookworm infection alongside the *S. mansoni* infection. Each slide from the same specimen was read by two different technicians, and eggs per slide were recorded. For quality control, 10% of the slides were randomly selected and re-examined by an independent experienced microscopist. No discrepancies were reported.

### 2.3. Anaemia Assessment and Anthropometric Measures

A finger-prick blood sample was taken from all the recruited children, and their haemoglobin (Hb) concentration (g/L) was measured using a portable HemoCue^®^ photometer (Ängelholm, Sweden). Anaemia was classified according to the World Health Organization guidelines [16] as non-anaemic (≥110 g/L), mildly anaemic (100–109 g/L), moderately anaemic (70–99 g/L), and severely anaemic (Hb < 70 g/L). Anthropometric parameters were taken to determine the nutrition status by measuring height, weight, and mid-upper arm circumference (MUAC) according to the WHO guidelines [17]. Body weight was measured to the nearest 0.1 kg using a portable digital scale (Seca Model 771, Seca GmbH, Hamburg; Germany). Height was measured barefooted to the nearest 0.1 cm using a portable stadiometer with an attached headboard. The age in months of each participant was recorded as reported by the parent or caretaker.

### 2.4. Morbidity Assessment

After Kato-Katz examinations, all *S. mansoni*-infected children and those who did not have any traces of *S. mansoni* eggs in the three stool samples were recruited into the study for morbidity assessment.

#### 2.4.1. Physical Examination

Abdominal palpations were performed independently by two experienced nurses on each child to detect pathological changes in the liver and spleen, which may be due to *S. mansoni* infection. The size of a palpable liver (left lobe and right lobe) was measured at the mid-sternal line (MSL) and right mid-clavicular line (MCL), whereas a palpable spleen was measured along the mid-axillary line (MAL). A liver edge and/or splenic tip extending ≥2 cm below the costal margins were considered enlarged. The consistency of each palpable organ was recorded as soft, firm, or hard. Liver tenderness and tipped spleen were also examined. The two nurses disclosed their measurements after each examination, and in case of any variations or discrepancy, re-examinations were performed with the aim of reaching a consensus.

#### 2.4.2. Ultra-Sonographic Examination

Abdominal ultrasonography was performed using a portable ultrasonographical device (Aloka^®^, SonocameraSSD-500 Tokyo, Japan) with a convex 3.5 MHz transducer, according to WHO standard guidelines [18]. Examinations were performed by a senior sonographer with extensive experience in *S. mansoni*-related ultrasonography. The children were examined lying on their backs with their legs stretched on an examination table. Measurements from the upper to the caudal margin in the left parasternal line (PSL) were done for the spleen length (SL) and the left liver lobe. The size of the right liver lobe was measured in the right mid-clavicular line (MCL), whereas the portal-vein diameter (PVD) was measured at the point where the portal vein enters the porta hepatica at the lower end of the caudate lobe. In children with texture patterns suggestive of periportal fibrosis (PPF), a picture was taken and the corresponding texture pattern score recorded. The sonographic picture of the liver texture was translated into six texture patterns (A–F), as described in WHO guidelines of the Niamey protocol [18]. Pattern A indicates normal liver texture, while patterns B–F indicate varying levels of liver fibrosis. Hepatomegaly, splenomegaly, and increased PVD were defined as two and four standard deviations (2SDs, 4SDs) above standard reference measurements for healthy uninfected children [19].

### 2.5. Ethical Considerations

The study was approved by the Research and Ethics Committee, College of Health Sciences, Makerere University, and cleared by the Uganda National Council of Science and Technology. Permission to conduct the study in the region was obtained from the President’s office/Residential District Commissioner. At the beginning of the study, caregivers were explained the objectives of the study in the local language and were asked to decide the participation of their children. Children aged 1–5 years were too young to understand the study objectives, but their caretakers gave informed permission for their child(ren)’s involvement in the study. Any child that showed some dissent behaviour (like crying and resisting) was excluded from the study. Study participants who were identified as positive for intestinal schistosomiasis were treated with 40 mg/kg praziquantel by a qualified nurse after clinical and ultrasound examinations.

### 2.6. Statistical Analysis

Data were double entered using EpiData^TM^ version 3.1 (The EpiData Association, Odense, Denmark) and Microsoft Excel^®^ spreadsheet software [20]. The duplicates were compared and corrected for data errors. Statistical analysis was carried out in Stata/IC release 12.0 (StataCorp; College Station, TX, USA). Intensity of infection (expressed in eggs per gram of stool, epg) was calculated by multiplying the mean for the six slides by a factor of 24. The eggs were found to be over-dispersed and were thus log-transformed and intensities reported as geometric mean intensity (GMI) of epg among infected children. *Schistosoma mansoni* intensities were classified according to WHO guidelines [17] as light: 1–99 epg, moderate: 100–399 epg, and heavy: ≥400 epg. Hookworm intensities were classified as light: 1–1999 epg, moderate: 2000–3999 epg, and heavy: ≥4000 epg. The nutritional status of children was assessed using four anthropometric indices: weight-for-height: (% wasting), weight-for-age: (% underweight), height-for-age: (% stunting), and MUAC: (% wasting/acute). These indices were derived as a standard deviation (SD) or Z-score of a child’s measurement according to the growth reference of the WHO-NCHS [17]. The Z-score of −2SD from the median is generally considered the cut-off point for screening children who are likely to be malnourished and then classified as ‘above normal’ if they were above or equal to Z-score of +2. Reference data were used to adjust the sizes of liver, spleen, and PVD for height [19]. The values for organ sizes, spleen length, PSL, and PVD were then classified as ‘normal’ if they were below or equal to the mean +2SD, ‘moderately abnormal’ if they were more than 2SD but below or equal to the mean +4SD, and ‘severely abnormal’ if they were above the mean +4SD. Liver texture pattern (LTP) A was considered to show no liver fibrosis, while B, C, D, E, and F indicated disease. Proportions were calculated and comparisons made using the Pearson chi-square (χ^2^) test. To assess whether *S. mansoni* infection was associated with morbidity indices, we performed analysis at two levels. First, we compared anthropometry measurements, level of anaemia, and size of spleen, liver, and PVD among children who were infected with those who were not infected. Secondly, we determined the association between potential morbidity indicators and *S. mansoni* intensity using a stepwise multiple regression analysis. Log-transformed egg counts (eggs per gram of faeces, epg) were also used for the linear regression model. *p*-Values of less than 0.05 were considered statistically significant.

## 3. Results

### 3.1. Parasitological Findings

Of the 916 included children, 686 (74.9%) were positive for *S. mansoni,* while 230 (25.1%) did not have any traces of *S. mansoni* eggs in the three stool samples. Of the 686 infected children, 397 (57.9%) were lightly infected, while 156 (22.7%) were moderately infected and 133 (19.4%) were heavily infected. The overall geometric mean intensity (GMI) among the infected children was 294.2 epg. A total of 71 children (7.8%) had hookworm infection, with 70 having light infection. Only one of the infected children had moderate hookworm intensity while none had heavy infection (Table 1).

### 3.2. Baseline Characteristics of the Infected and Uninfected Children

Most children—and consequently most infected children—came from Mayuge and Namayingo districts, while the other districts contributed fewer children (Table 1). Mayuge and Jinja districts had the highest (51.2%) and lowest (2.2%) number of infected children, respectively. Sex and hookworm infection were not associated with *S. mansoni* infection (*p* >0.05). Older preschool-aged children (49–60 months) were more often infected with *S. mansoni* compared to younger ones (12–48 months), as shown in Table 1.

### 3.3. Anaemia, Anthropometric Indicators, and Liver Texture Patterns

All 916 children had data on haemoglobin (Hb) concentration, anthropometry, and ultrasonography collected. Compared to the uninfected, children who were infected with *S. mansoni* were more likely to be anaemic (Table 2).

Thus, 52.4% of the infected children were anaemic (mild, moderate, and severe anaemia combined) compared to 35.3% of the uninfected children; *p <* 0.0005. Overall, 97.5%, 84.1%, 91.2%, and 95.4% of the participating children had normal weight-for-age, height-for-age, weight-for-height, and MUAC for age, respectively (Table 2). There was no significant difference in underweight, stunting, and severe wasting between infected and uninfected children, but moderate wasting was significantly (*p =* 0.001) more often found in the uninfected children compared to the infected ones (Table 2). Children who were infected with *S. mansoni* were more likely to be moderately malnourished compared to the uninfected ones (5.5% vs. 1.3%; *p =* 0.007). More *S. mansoni*-infected children were observed with liver texture abnormalities compared to uninfected children (14.7% vs. 3.5%; *p* < 0.0005) as shown in Table 2. Abnormal LTP scores ranged from pattern B0 (6.1%) to pattern C2 (0.3%). The uninfected children were identified with liver patterns A (normal, 96.5%) and B0 (feather streaks, 3.5%). Liver texture patterns that indicate more advanced liver damage (B1–C2) were found only in the *S. mansoni*-infected children (Table 2).

### 3.4. Schistosoma mansoni Effected Organ Enlargement among Preschool-Aged Children

Table 3 summarizes clinically detected spleen and liver enlargement among *S. mansoni*-infected and uninfected children.

*Schistosoma mansoni* uninfected children had a higher rate of palpable spleen (36.1%) compared to the infected (19.2%); this difference was statically significant (*p <* 0.0005). Uninfected children had soft spleens significantly (*p* < 0.0005) more often (28.7%) compared to the infected ones (10.5%). There was a significant difference in liver palpability between *S. mansoni* infected and uninfected children, 17.2% vs. 10.8%; *p =* 0.02. Infected children had firm livers significantly (*p <* 0.005) more often (3.2%) compared to the uninfected ones (0.9%). Table 4 summarizes spleen and liver enlargement among *S. mansoni* infected and uninfected children as detected by ultrasonography.

**Figure 1:**
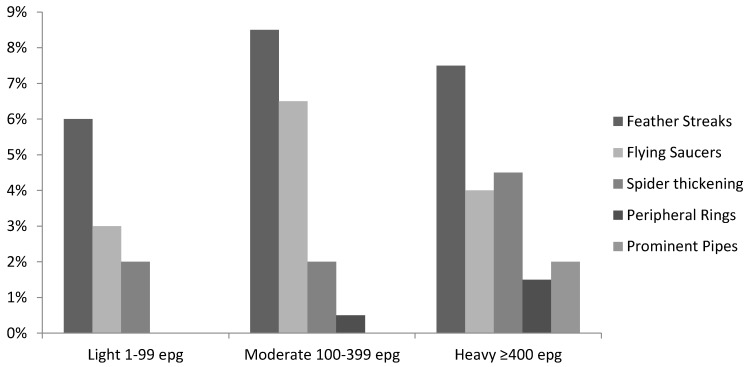
Liver fibrosis measured by ultrasound associated with *S. mansoni* infection intensity in preschool-aged children.

Thus, a significantly higher proportion of uninfected children had normal organs compared to *S. mansoni*-infected ones (29.1% vs. 15.5%, *p* = 0.005). Marked splenic length (SL) exceeding mean +4SD of height-adjusted reference values was observed in 31.5% and 13.9% of infected and uninfected children, respectively (*p* < 0.0005). Uninfected children had a significantly (*p* = 0.005) higher number of moderately lengthened spleens (27.8%) compared with 16.8% found in the infected children. The left liver lobe was significantly more often (*p* < 0.0005) much enlarged (mean +4SD) in the infected children (26.2%) than in the uninfected children (10.4%). In contrast, the right liver lobe was significantly shrunken in a higher percentage of the uninfected children compared to the infected ones (2.6% vs. 0.9%; *p* = 0.04). The portal vein diameter was found significantly (*p* = 0.02) dilated only in the infected children. There was a strong positive relationship between liver texture patterns and *S. mansoni* infection intensity among the preschool-aged children (Table 5, Figure 1); liver damage increased with increase in intensity of infection (R^2^ = 0.029, Coef: 0.06, *p* < 0.001).

The children with advanced liver damage of prominent pipe stems (liver pattern C2) had an abnormally high mean intensity of infection compared to those with other liver texture patterns (Table 5). Lightly infected children were observed with liver patterns B0, B1, and B2, while liver pattern C2, prominent pipe stems, associated with advanced liver damage, was observed in the heavily infected children only (Figure 1).

**Figure 2:**
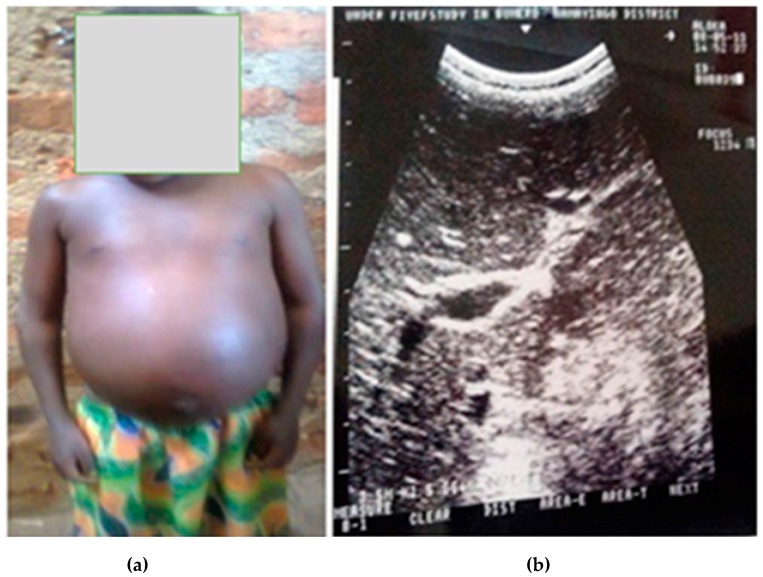
Liver fibrosis (**a**) with prominent pipe stems (**b**) in a preschool-aged child.

Ultrasound examination showed detectable liver fibrosis (Figure 2a,b) in a young girl of 5 years from Namayingo district, Bumeru landing site. There were no cases of ascites detected.

### 3.5. Relationship between S. mansoni Infection Intensity and Morbidity Indicators among Preschool-Aged Children

In a stepwise multiple regression analysis with *S. mansoni* infection intensity as the dependent variable (Table 6), age of the child, anaemia, liver texture pattern, and size of the left liver lobe were significantly associated with *S. mansoni* intensity (*p* < 0.05).

For every one-year (12 months) increase in the age of the child there was an increase in *S. mansoni* intensity of infection by 0.33 epg. For every unit increase in anaemia (normal, mild, moderate, severe), there was an increase in *S. mansoni* intensity of infection by 0.12 epg. For every advancement in the liver pattern (A to C) there was an increase in *S. mansoni* intensity of infection by 0.1 epg. For each unit increase in the size of the left liver lobe, there was an increase in *S. mansoni* intensity of infection by 0.12 epg. The size of the right liver lobe, spleen length, MUAC for age, weight-for-height, height-for-age, weight-for-age, district of origin, and sex were not associated with *S. mansoni* intensity of infection and were excluded from the model.

## 4. Discussion

Morbidity associated with schistosomiasis was detected in preschool-aged children aged 12–60 months using both clinical and ultrasound examination. However, sonographic examinations revealed much more disease related to infection with *S. mansoni* than was detected by physical palpation. This is in accordance with earlier studies, which also showed that ultrasound techniques in comparison to clinical examinations provide more sensitive and precise measurements of liver and spleen enlargement associated with *S. mansoni* infections [21,22,23]. Additionally, advanced degrees of *S. mansoni*-related liver fibrosis and splenomegaly are more easily detected by ultrasound than by abdominal palpation [21,24,25,26]. According to our knowledge, there are no studies that have assessed morbidity in children less than six years of age infected with *S. mansoni*, but studies have revealed that preschool-aged children get exposed to *S. mansoni*-infested waters through bathing and playing [5]. On the other hand, other studies on school children older than six years found no significant difference between *S. mansoni*-infected and -uninfected school children with respect to liver and spleen enlargement [11,27].

The present study shows that much of the morbidity detected in children aged 12–60 months is attributed to *S. mansoni* infection, and it is measured through liver size enlargement—especially the left liver lobe. Enlargement of the left liver lobe is a pattern related to infection with *S. mansoni* [28], and is usually associated with acute schistosomiasis [13,29,30], especially in childhood [9,31]. This may explain why preschool-aged children in this study who may have experienced their first infection recently had more enlarged livers compared to spleens. This is further explained by the finding of the left liver lobe being significantly firmer, and thus palpable, in some of the children that were infected with *S. mansoni*. However, it is well known that intestinal schistosomiasis is second only to malaria in causing morbidity [32], and that the two diseases are co-endemic in sub-Saharan Africa. Malaria can therefore be a confounder (especially for spleen enlargement), and unfortunately the malaria infection status of the included children was not investigated.

This study further reveals that *S. mansoni*-related morbidity increases with increase in intensity of infection. Children with advanced liver damage of prominent pipe stems, for instance, are the same children that were found with an abnormally high mean intensity of infection. Other morbidity indicators followed suit, showing increased *S. mansoni* intensity associated with increased morbidity. This study further shows that older children (49–60 months) are more likely to be infected with *S. mansoni* than their younger counterparts (12–48 months), and are hence at a higher risk of increased intensity of infection and morbidity development. It is therefore not surprising that advanced liver damage that occurs after a long exposure to *S. mansoni* infection was identified in a 60-month-old child in this study. This condition is related to high parasite burden with the production of numerous eggs, as described in other studies [33]. Other studies have also shown that the intensity of *S. mansoni* infection in preschool-aged children increases with age [5,6,34].

Few children (<1%) infected with *S. mansoni* in this study had detectable periportal fibrosis (PPF). This is the first study to report Symmer’s pipe stem fibrosis in children less than six years of age. This severe liver morbidity is usually observed in long-standing infection with *S. mansoni* [35]. Our results may suggest that with extremely heavy infection with *S. mansoni,* the period from infection to development of PPF may be reduced. Dilation of the portal vein was found in 2.3% of children infected with *S. mansoni*, and none of the uninfected ones. The size of PVD has been shown to increase linearly with intensity of infection with *S. mansoni* and with the size of the spleen [8,31,36,37,38]. This is expected, as spleen enlargement is known to contribute more blood at a high pressure to the portal system [39,40], and the portal pressure created leads to dilation of the portal vein [41].

Results in the present study indicate that *S. mansoni*-infected children had lower haemoglobin levels than the egg-negative children. This is consistent with conventional knowledge that *S. mansoni* is one of the myriad causes of anaemia [42,43]. Although known to cause anaemia [42,43], high prevalence of hookworm infection was not found in the present study, and intensity of infection was relatively low. These results are not surprising because hookworm infection is usually not prevalent in this age group—it is more prevalent in older children and adults [20]. Moreover, hookworm infection was observed in equal proportions in *S. mansoni* infected and uninfected children, and yet anaemia was significantly associated with *S. mansoni* infection.

The relationship between nutritional status and infection with *S. mansoni* showed a significant rate of malnutrition (MUAC for age) among infected children. Other studies in school-age children found a similar positive correlation between *S. mansoni* infection and malnourished children [44,45]. Unexpectedly, the proportion of underweight or wasted (mean weight-for-height) children was significantly higher among *S. mansoni*-negative children in this study. This is in contrast with other studies [28], which have shown that weight loss is one of the consequences of infection with *S. mansoni* when egg production starts. This indicates that there are causes of malnutrition other than *S. mansoni* infection in preschool-aged children; for instance, it could be caused by other illnesses or lack of adequate and proper nutrition. A similar finding was observed in school-age children [45]. Malnutrition in this age group could also be attributed to *Ascaris lumbricoides* infection as indicated in some studies [4], but there were no *Ascaris* eggs detected in the examined stool in this study.

Our study had strengths that lead credence that infection with *S. mansoni* may contribute to the causation of the morbidity indicators served. First, the association between *S. mansoni* and liver enlargement was observed more often with the left lobe compared to the right, and this is consistent with conventional knowledge that *S. mansoni* is more likely to affect the left lobe more than the right lobe. Secondly, the dilation of the portal vein was observed only in children who were infected with *S. mansoni*. Thirdly, the severity of morbidity (anaemia, liver size, and PVD) increased with intensity of infection with *S. mansoni*, thereby indicating a dose-related association that is more linked to causation than merely association [1]. Finally, the association between morbidity and severity of infection remained after controlling for known cofounders.

Nevertheless, some limitations still remain, and caution is needed in interpreting our findings. We did not measure co-infection with malaria, which is common in the study area, and is a known predictor of some of the morbidity indicators assessed [46,47,48]. We did, however, adjust for age and place of residence (district), which has a high influence on infection with malaria. Furthermore, this was a cross-sectional study which is useful for investigating associations, but is more problematic in investigating cause-relationships. Thus, longitudinal follow-up studies are needed to monitor changes in indicators as schistosomiasis is controlled.

In conclusion, the present study reveals that *S. mansoni*-associated morbidity is common among preschool children aged 12 to 60 months. Further, the study shows that organ enlargement as a result of *S. mansoni* infection in preschool-aged children is mainly due to liver fibrosis and not splenomegaly. These children should be a target of the schistosomiasis control program and should be treated with praziquantel [2,4].

## Figures and Tables

**Table 1:** Background characteristics of *S. mansoni* infected and uninfected preschool-aged children.

	Uninfected Children		Infected Children		
Variable	*n* = 230	% (95% CI)	*n* = 686	% (95% CI)	*p*-Value *
District of residence					
Bugiri	13	5.7 (2.7–8.7)	24	3.5 (2.1–5.0)	0.15
Buikwe	6	2.6 (0.5–4.7)	38	5.5 (3.8–7.2)	0.072
Jinja	10	4.3 (1.7–7.0)	15	2.2 (1.1–3.3)	0.082
Mayuge	62	27.0 (21.3–32.7)	351	51.2 (47.5–55.0)	<0.0005
Namayingo	139	60.4 (54.1–66.7)	258	37.6 (34.0–41.2)	<0.0005
Sex					
Male	114	48.3 (41.8–54.8)	347	50.1 (46.4–53.8)	0.62
Female	116	51.7 (45.2–58.2)	339	49.9 (46.2–53.6)	0.62
Hookworm infection					
None	209	90.9 (87.2–94.6)	636	92.7 (90.8–94.7)	0.37
Light (1–1999 epg)	20	8.7 (5.1–12.3)	50	7.3 (5.4–9.3)	0.49
Moderate (2000–3999 epg)	1	0	0	0	
Heavy (≥4000 epg)		0	0	0	
Age in months					
12–24	4	2.2 (0.3–4.1)	19	2.6 (1.4–3.8)	0.71
25–36	82	35.7 (30.0–42.0)	129	18.8 (16.0–21.7)	<0.0005
37–48	81	34.8 (28.6–41.0)	211	30.9 (27.4–34.6)	0.28
49–60	75	27.4 (21.6–33.2)	315	47.7 (44.0–51.4)	<0.0005

* Pearson chi-square tests; epg: eggs per gram of faeces; CI: confidence interval.

**Table 2:** Anaemia, nutrition status and liver texture among *S. mansoni* infected and uniinfected children.

Variable	Uninfected Children (230)	Infected Children (686)	*p*-Value *
	*n* (%)	*n* (%)	
Anaemia (Hb Concentration)			
Normal (110–150 g/L)	149 (64.7)	327 (47.6)	<0.0005
Mild (100–109 g/L)	37 (16.1)	161 (23.5)	0.019
Moderate (70–99 g/L)	39 (17.0)	187 (27.3)	0.001
Severe (<70 g/L)	5 (2.2)	11 (1.6)	0.57
Weight-for-age			
Normal	222 (96.5)	671 (97.8)	0.28
Moderate	7 (3.0)	13 (1.9)	0.31
Severe (underweight)	1 (0.4)	2 (0.3)	0.74
Height-for-age			
Normal	193 (83.9)	577 (84.1)	0.94
Moderate	27 (11.7)	60 (8.7)	0.18
Severe (stunted)	10 (4.3)	49 (7.1)	0.14
Weight-for-height			
Normal	197 (85.7)	638 (93.0)	0.001
Moderate	15 (6.5)	14 (2.0)	0.001
Severe (wasted)	18 (7.8)	34 (5.0)	0.10
MUAC for age			
Normal	227 (98.7)	647 (94.3)	0.006
Moderate	3 (1.3)	38 (5.5)	0.007
Severe (malnourished)	0 (0)	1 (0.2)	0.56
Liver texture pattern (LTP)			
Normal (A)	222 (96.5)	585 (85.3)	<0.0005
Feather streaks (B0)	8 (3.5)	48 (7.0)	0.078
Flying saucers (B1)	0 (0)	28 (4.1)	<0.001
Spider thickening (B2)	0 (0)	19 (2.8)	0.006
Peripheral rings (C1)	0 (0)	3 (0.4)	0.32
Pipe stems (C2)	0 (0)	3 (0.4)	0.32

* Pearson chi-square tests. Hb: haemoglobin; MUAC: mid-upper arm circumference.

**Table 3:** Clinically detected liver and spleen enlargement among *S. mansoni* infected and uninfected children.

Variable	Uninfected Children (*n* = 230) % (95% CI)	Infected Children (*n* = 686) % (95% CI)	*p*-Value *
**Organ Palpability**			
Normal spleen	63.9 (57.9–70.1)	80.8 (77.9–83.8)	<0.0005
Palpable spleen	36.1 (29.9–42.3)	19.2 (16.3–22.2)	
Normal liver	89.1 (85.1–93.1)	82.8 (80.0–85.6)	0.02
Palpable liver	10.8 (6.8–14.3)	17.2 (14.2–20.0)	
**Liver Consistency**			
Normal	90.0 (86.1–93.9)	82.8 (79.3–85.1)	0.008
Soft	9.1 (5.4–12.8)	13.8 (11.2–16.4)	0.06
Firm	0.9	3.2 (1.9–4.5)	0.0007
Hard	0	0.1	0.74
**Spleen Consistency**			
Normal	65.2 (54.0–71.4)	80.3 (77.3–83.3)	<0.0005
Soft	28.7 (22.9–34.6)	10.5 (8.2–12.8)	<0.0005
Firm	5.2 (2.3–8.1)	8.2 (6.2–10.3)	0.14
Hard	0.9	1.0	0.84

* Pearson chi-square tests; CI: confidence interval.

**Table 4:** Ultrasound detected liver and spleen enlargement among *S. mansoni* infected and uninfected children.

Variable	Uninfected Children (*n* = 230) % (95% CI)	Infected Children (*n* = 686) % (95% CI)	*p*-Value *
**Organomegaly**			
Normal	29.1 (23.2–35.0)	15.5 (10.8–20.18)	0.005
Hepatomegaly	30.0 (24.1–36.0)	35.8 (29.6–42.0)	0.11
Splenomegaly	25.2 (19.6–30.8)	30.6 (24.6–36.6)	0.12
Hepatosplenomegaly	15.7 (11.0–20.4)	18.1 (13.1–23.1)	0.40
**Splenic Length**			
Normal (+≤2SD)	58.3 (51.9–64.7)	51.7 (45.2–58.2)	0.08
Moderate length (+>2SD to ≤4SD)	27.8 (22.0–33.6)	16.8 (12.0–21.6)	0.005
Marked length (+4SD)	13.9 (9.4–14.4)	31.5 (25.5–37.5)	<0.0005
**Left Liver Lobe Enlargement**			
Normal (+≤2SD)	57.0 (50.6–63.4)	49.3 (42.8–55.7)	0.04
Enlarged (+>2SD to ≤4SD)	32.6 (26.5–38.7)	24.5 (19–30.1)	0.02
Much Enlarged (+4SD)	10.4 (6.5–14.4)	26.2 (20.5–31.9)	<0.0005
**Right Liver Lobe Enlargement**			
Normal (+≤2SD)	97.4 (95.3–99.5)	99.1 (97.9–100)	0.04
Shrunk (+>2SD to ≤4SD)	2.2 (0.3–4.1)	0.9	0.12
Severely Shrunk (+4SD)	0.4	0	0.08
**Portal Vein Diameter (PVD)**			
Normal (+≤2SD)	100	97.7 (95.8–99.6)	0.02
Dilated (+>2SD to ≤4SD)	0	2.3 (0.4–4.2)	0.06
Marked Dilation (+4SD)			

* Pearson chi-square tests; SD: standard deviation; CI: confidence interval.

**Table 5:** *Schistosoma mansoni* geometric mean intensity and liver texture pattern among infected preschool-aged children.

Liver Texture Pattern	Geometric Mean Infection Intensity (epg)	95% CI	*p*-Value *
Normal	274.7	233.4–316.0	<0.001
Feather streaks (B0)	344.2	176.3–512.0	
Flying saucers (B1)	225.3	131.0–319.6	
Spider thickening (B2)	390.5	156.1–625.0	
Peripheral rings (C1)	432.0	190.2–673.8	
Pipe stems (C2)	3202.7	1747.6–4657.7	

* Pearson correlation coefficient tests; CI: confidence interval.

**Table 6:** Regression coefficients, 95% confidence intervals (95% CI), and corresponding *p*-values of variables found to be significantly associated with *S. mansoni* infection intensity (epg) among preschool-aged children in a multivariable linear regression model.

Variable	Regression Coefficient (95% CI)	*p*-Value
Age (months) ^a^	0.33 (0.26–0.40)	<0.0005
Anaemia ^b^	0.12 (0.05–0.19)	<0.0005
Liver texture pattern ^c^	0.10 (0.01–0.19)	0.021
Left liver lobe enlargement ^d^	0.12 (0.04–0.20)	0.003

*n* = 900, overall adjusted R^2^ = 0.11, overall *p* < 0.0005. ^a^ Coded as 1 = 12–24 months; 2 = 25–36 months; 3 = 37–48 months; 4 = 49–60 months. ^b^ Coded as 0 = normal; 1 = mild anaemic; 2 = moderate anaemic; 3 = severe anaemic. ^c^ Coded as 0 = normal; 1 = feather streaks; 2 = flying saucers; 3 = spider thickening; 4 = prominent peripheral rings; 5 = prominent pipe stems. ^d^ Coded as 0 = normal; 1 = enlarged; 2 = much enlarged.
